# Cytomegalovirus prophylaxis with letermovir in pediatric (birth to <18 years of age) hematopoietic cell transplant recipients: pharmacokinetics, efficacy, and safety results of a Phase 2b study

**DOI:** 10.1128/aac.00420-25

**Published:** 2025-08-18

**Authors:** Andreas H. Groll, Lara Danziger-Isakov, Aharon Gefen, Christopher J. Fraser, Johannes H. Schulte, Bella Bielorai, Nicole A. Karras, David Bueno, Peter J. Shaw, Natalya Broyde, Barbara Haber, Christopher L. Gilbert, Mayankbhai Patel, Jacqueline B. McCrea, Cyrus Badshah

**Affiliations:** 1 University Children’s Hospital Münster, Münster, Germany; 2 Cincinnati Children’s Hospital Medical Center, University of Cincinnati, Cincinnati, Ohio, USA; 3 Ruth Rappaport Children’s Hospital, Rambam Health Care Campus 58878, Haifa, Israel; 4 Faculty of Medicine, Technion Israel Institute of Technology 26747, Haifa, Israel; 5 Queensland Children’s Hospital, Brisbane, Queensland, Australia; 6 Charité Universitätsmedizin Berlin 14903, Berlin, Germany; 7 Sheba Medical Center 26744, Tel HaShomer, Israel; 8 Faculty of Medicine, Tel Aviv University 26745, Tel Aviv, Israel; 9 City of Hope Cancer Center, Duarte, California, USA; 10 Hospital Infantil Universitario La Paz 542070 , Madrid, Spain; 11 Royal Alexandra Hospital for Children, Westmead, New South Wales, Australia; 12 Merck & Co., Inc., Rahway, New Jersey, USA; IrsiCaixa Institut de Recerca de la Sida, Barcelona, Spain

**Keywords:** pharmacokinetics, letermovir, cytomegalovirus, hematopoietic cell transplantation, pediatric

## Abstract

Letermovir, a cytomegalovirus (CMV) terminase complex inhibitor, was first approved for prophylaxis of CMV infection and disease in adult CMV-seropositive allogeneic hematopoietic cell transplant (HCT) recipients (R+). This study evaluated the pharmacokinetics (PK), efficacy, and safety of letermovir in pediatric R+ allogeneic HCT recipients. In this Phase 2b, single-arm, open-label study, 65 participants were enrolled sequentially in three age groups (AG; AG1, 12 to <18 years; AG2, 2 to <12 years; and AG3, birth to <2 years). PK was evaluated in an initial cohort in each AG using intensive PK data to confirm or modify dosing before enrolling the remaining participants. Adult HCT population PK (PopPK) data were used to establish the exposure reference range. The adult letermovir dose evaluated in AG1 and AG2 participants achieved exposures generally within the adult HCT reference range. In AG3, the initial cohort (letermovir with cyclosporin A) achieved exposures trending lower than the median exposure target; the letermovir dose was increased for the remaining participants. Efficacy and safety in pediatric participants were generally consistent with adult HCT data. A pediatric HCT PopPK model was developed to determine dose recommendations to be included in patient prescribing information. The doses evaluated achieved exposures generally within the adult HCT reference range. At exposures achieved, letermovir was efficacious and safe in preventing clinically significant CMV infection in pediatric allogeneic HCT recipients. The observed concentration data informed a pediatric PopPK model to optimize final letermovir dose recommendations in this population.

**CLINICAL TRIALS:**

This study is registered with ClinicalTrials.gov as NCT03940586.

## INTRODUCTION

Cytomegalovirus (CMV) is a common viral pathogen associated with considerable morbidity and mortality in recipients of allogeneic hematopoietic cell transplant (HCT), with CMV-seropositive recipients (R+) being at the highest risk of CMV reactivation (1). In Europe in 2021, ~20% of recipients of allogeneic HCTs were pediatric patients, with over 4,000 patients aged <18 years receiving an HCT that year (2). More than 12,000 HCTs, or approximately 10% of total HCTs, in the United States were performed in pediatric patients (aged <18 years) during 2016–2020 (3). Approximately one-quarter of pediatric allogeneic HCT recipients develop CMV infection (i.e., CMV viremia) (4). There were no approved antiviral agents available for CMV prophylaxis in pediatric allogeneic HCT recipients when the current study was initiated. Therefore, there was a substantial unmet need for a safe and effective agent for CMV prophylaxis in this population.

Letermovir, a CMV DNA terminase inhibitor, is approved for the prophylaxis of CMV infection and disease in adult R+ allogeneic HCT recipients in over 60 countries, including the United States, the European Union, and Japan (5). Results of a Phase 3 placebo-controlled study (P001, NCT02137772) conducted in adult R+ allogeneic HCT recipients demonstrated that letermovir was generally safe, well tolerated, and efficacious in the prevention of clinically significant CMV infection (CS-CMVi; defined as initiation of anti-CMV pre-emptive therapy [PET] based on documented CMV viremia and/or the onset of CMV end-organ disease) (6). In adult HCT recipients (P001), exposure-response analyses indicated efficacy within the entire range of exposures achieved with letermovir 480 mg once daily, adjusted to 240 mg once daily with concomitant cyclosporin A (CsA). The pathogenesis of CMV infection (viremia) and disease, and the mechanism of action of letermovir, are not anticipated to differ in pediatric patients compared with adults. Therefore, letermovir pediatric exposures within the range of exposures achieved in adult HCT recipients are expected to provide comparable efficacy and safety in the pediatric population. The steady-state area under the concentration-time curve from 0 to 24 h post-dose (AUC0–24) in adults following oral and intravenous (IV) letermovir 480 mg (without CsA) was defined as the pediatric HCT exposure target (reference exposure range, 16,900 to 148,000 h × ng/mL).

The objectives of this study were to evaluate the pharmacokinetics (PK), efficacy, and safety of letermovir in pediatric R+ allogeneic HCT recipients. The PK data from this study were used to assess plasma exposures following the doses evaluated and to develop a pediatric HCT population PK (PopPK) model to derive the final dose recommendations for approval in the pediatric HCT population (7).

## MATERIALS AND METHODS

### Study design and participants

**Fig 1:**
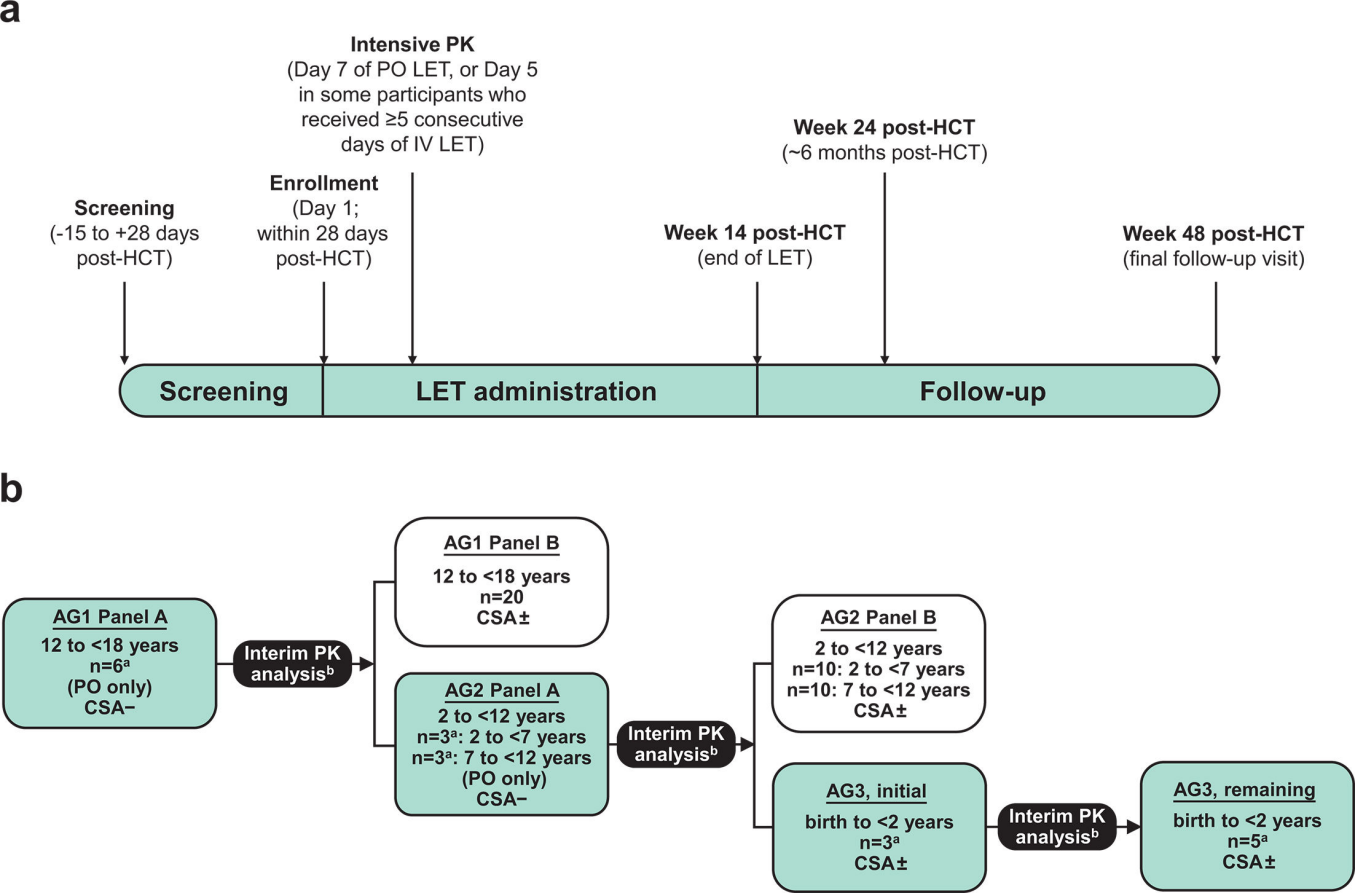
(**a**) Study design and (**b**) sequential pharmacokinetic (PK) evaluation of age groups. AG, age group; CSA±, may receive concomitant cyclosporin A; CSA−, cannot receive concomitant cyclosporin A; HCT, hematopoietic cell transplant; IV, intravenous; LET, letermovir; PO, oral. Intensive PK sampling was performed pre-dose and 1, 2.5, 8, and 24 h post-dose. Sparse PK sampling took place at Weeks 2, 4, 6, 8, 10, 12, and 14 post-HCT during the treatment phase. ^a^Number of PK-evaluable participants. ^b^Interim PK analysis occurred at three time points: when all evaluable participants had completed intensive PK sampling in AG1 Panel A, when all evaluable participants had completed intensive PK sampling in AG2 Panel A, and when the first three evaluable participants had completed intensive PK sampling in AG3.

This Phase 2b (P030, NCT03940586), open-label, single-arm study assessed PK, efficacy, and safety of letermovir for CMV prophylaxis in pediatric participants from birth to <18 years of age at risk of developing CMV infection and/or disease following an allogeneic HCT. The study design is summarized in Fig. 1a. All study participants received letermovir within 28 days post-HCT through Week 14 post-HCT. Thereafter, participants were followed through Week 24 post-HCT for efficacy, and through Week 48 post-HCT for safety and tolerability. Intensive and sparse PK sampling were performed to characterize letermovir PK in pediatric HCT recipients (Fig. 1a).

There were three interim PK analyses planned to confirm dosing. Interim analysis 1 for an initial cohort of participants for age group (AG) 1 Panel A (aged 12 to <18 years, *n* = 6) was used to determine the optimum dosing for oral letermovir in the absence of CsA. The final dose selected in AG1 Panel A simultaneously triggered enrollment of AG1 Panel B and AG2 Panel A. Similarly, with interim analysis 2, AG2 Panel A (aged 2 to <7 years, *n* = 3; and aged 7 to <12 years, *n* = 3) PK was evaluated to determine the optimum oral letermovir dosing (without concomitant CsA). The final dose selection in AG2 Panel A simultaneously triggered enrollment of AG2 Panel B and AG3 (aged birth to <2 years; initial cohort, *n* = 3). Interim analysis 3 informed dosing for the remaining cohort of AG3 (*n* = 5). All AG3 participants (initial cohort, *n* = 3 and remaining participants, *n* = 5) were dosed with oral or IV letermovir, with or without CsA coadministration, prior to PK evaluation (Fig. 1b).

Interim analysis data for AG1 were previously reported and demonstrated that administration of letermovir 480 mg once daily (240 mg once daily with concomitant CsA) in adolescents resulted in exposures within the prespecified bounds of the adult HCT exposure range, as well as an efficacy and safety profile similar to adults (8). The study was subsequently completed on 25 August 2023, and final analyses of the data for the entire study population are presented here.

Eligible participants were recipients of a first allogeneic HCT (bone marrow, peripheral blood stem cell, or cord blood) within 28 days prior to enrollment and had undetectable CMV DNA from a plasma or whole blood sample collected within 5 days prior to enrollment. AG1 participants had to be documented with a CMV R+ status within 90 days prior to enrollment; AG2/3 participants had to be documented as CMV R+ within 90 days prior to enrollment and/or the donor had to be CMV-seropositive within 1 year prior to enrollment. Given the relatively small number of HCTs performed in pediatric patients and the lower CMV seroprevalence in younger individuals (3, 4), restricting enrollment to only include participants with CMV R+ status in this study would have been challenging, particularly in the youngest AGs. These considerations were taken into account when designing the study to enable broad enrollment across the subpopulation of HCT recipients. Key exclusion criteria included CMV end-organ disease within 6 months prior to enrollment, prior allogeneic HCT, and evidence of CMV viremia at any time between informed consent or HCT procedure (whichever was earlier) until enrollment. Full eligibility criteria are provided in the Supplementary Material (Tables S1 and S2).

Participants’ legally acceptable representative(s) provided written informed consent. The study was reviewed and approved by the appropriate institutional review board or independent ethics committee at each center. This study was conducted in accordance with local and/or national regulations (including all applicable protection laws and regulations), the International Council for Harmonization of Technical Requirements for Pharmaceuticals for Human Use Good Clinical Practice, and the ethical principles that have their origin in the Declaration of Helsinki regarding independent ethics committee review, informed consent, and the protection of human participants in biomedical research.

### Letermovir dose selection and administration

Initial dose selection was guided by both physiologically based PK and PopPK models. The pre-study initial dosing selections were based on simulations using an allometrically scaled adult HCT PopPK model. The participants in AG1 received the recommended daily adult dose of 480 mg letermovir (adjusted to 240 mg with concomitant CsA administration, if needed, in Panel B) through Week 14 post-HCT (without weight limits). The remaining initial dose selections for AG2 and AG3 were based on weight-band dosing. Dose modifications based on the interim analyses are described in the results (Table S3).

Participants received letermovir from the day of enrollment through Week 14 post-HCT (Fig. 1a). All Panel A participants in AG1 and AG2 received oral letermovir, with no CsA, from Day 1 through Day 7, after which they could receive letermovir by oral or IV administration. AG1/2 Panel B participants and AG3 participants received either oral or IV letermovir with or without concomitant CsA. Oral letermovir administration was preferred, with IV administration provided to participants who could not tolerate oral intake (e.g., due to nausea and vomiting, mucositis, or a malabsorptive condition). For oral administration, AG1 participants received either the currently marketed oral tablets or a newly developed oral granule formulation (or “oral pellets”), which were administered mixed with soft food or via a gastric or nasogastric tube; AG2 and AG3 study participants received only the oral granule formulation.

### PK exposure targets

The steady-state median target range for AUC0–24, predicted in adult HCT recipients from the Phase 3 PopPK model following administration of letermovir 480 mg orally or IV once daily without CsA, was 34,400–100,000 h × ng/mL (9). The lower and upper bounds of the adult HCT exposure range were 16,900 h × ng/mL (5th AUC0–24 percentile following letermovir 480 mg orally) and 148,000 h × ng/mL (95th AUC0–24 percentile following letermovir 480 mg IV), respectively.

### Objectives and end points

The primary objective of the study was to evaluate letermovir PK in pediatric participants grouped by standard age cohorts. PK end points were assessed in the per-protocol population (defined as the subset of participants who comply with the protocol sufficiently to ensure that these data are likely to exhibit the effects of the study drug, according to the underlying scientific model). The primary measure of PK exposure was the AUC0–24 for determination of letermovir exposure. This parameter was used to match pediatric exposure to the adult HCT reference range and is the focus of the results.

Efficacy was a secondary objective evaluated in all participants who received at least one dose of study drug and had no detectable CMV DNA on Day 1 when the study drug was initiated (primary efficacy population). The efficacy end points were the proportion of participants with CS-CMVi (1) through Week 14 and (2) through Week 24 post-HCT. Efficacy was assessed through Week 14, the period of the highest risk of CMV reactivation (1). Participants were followed for an additional 10 weeks, through Week 24, to assess rebound CMV infection after cessation of prophylaxis. CS-CMVi was defined as the initiation of anti-CMV PET based on documented CMV viremia and the participant’s clinical condition, and/or the onset of CMV end-organ disease. Documented viremia was defined as any detectable CMV viral DNA (including polymerase chain reaction results above or below the lower limit of quantification) using local laboratory testing. The primary missing data approach for the primary efficacy analyses was the non-completer = failure (NC = F) approach. Under this approach, not only were participants who developed CS-CMVi (true failures) counted as failures, but participants who prematurely discontinued from the study or who had missing outcome data were also imputed as failures. An alternative missing data approach, the Data as Observed (DAO) approach, was also used for the efficacy analyses. With this approach, participants who discontinued prematurely or had missing outcome data (i.e., imputed failures) were excluded from the analysis. The DAO approach thus accounts for the incidence of CS-CMVi (i.e., true failure rate) only.

Safety and tolerability were assessed by clinical review of relevant parameters, including adverse events (AEs), prospectively collected during the study. All AEs were collected through 28 days following the last dose of the study drug (treatment phase). Thereafter, only drug-related serious AEs (SAEs) and SAEs leading to death were collected through Week 48 post-HCT. The primary safety population consisted of all participants who received at least one dose of the study drug.

To assess genotypic resistance, CMV DNA sequence analysis was performed using next-generation sequencing on samples from participants with CS-CMVi. Letermovir resistance was assessed by genotypic analysis of the CMV terminase complex genes (UL56, UL89, and UL51) with detectable CMV DNA.

### Statistical analysis

 There was no formal hypothesis testing in this study. A noncompartmental analysis was used for the primary PK parameters of interest, which was AUC0–24 derived from the intensive PK sampling on Day 7. The interim noncompartmental PK analyses occurred at three intervals: (i) when all evaluable participants completed intensive PK sampling in AG1 Panel A; (ii) when all evaluable participants completed intensive PK sampling in AG2 Panel A; and (iii) when the first three evaluable participants completed intensive PK sampling in AG3. The final noncompartmental PK analyses were performed on the PK population who underwent intensive PK sampling. Individual AUC0–24 values were plotted by dose and weight. Additional PK parameters, including but not limited to *C*_max_, *T*_max_, and *T*_1/2_, were estimated as well, although they are not presented in this paper as AUC0–24 was the primary parameter determining dose selection.

For efficacy analyses, the estimated proportion of participants with CS-CMVi through Weeks 14 and 24 post-HCT, 95% CIs were calculated based on the exact binomial method of Clopper and Pearson (10). For safety analyses, the proportions of participants within different categories of AEs were summarized, with 95% CIs estimated by the Clopper-Pearson method (10).

## RESULTS

### Participants

**Fig 2:**
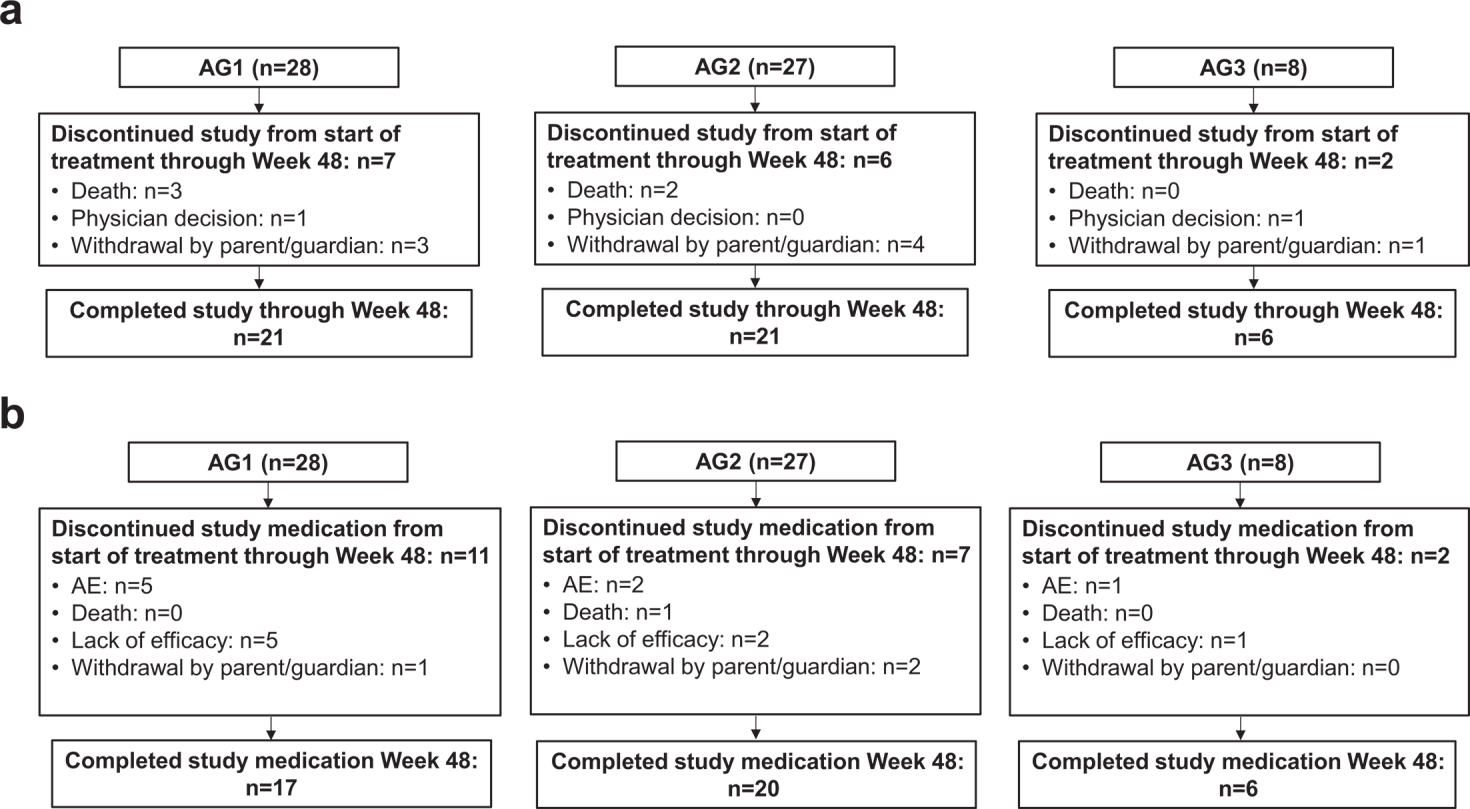
Participant disposition (all randomized and treated participants): (**a**) status for trial and (**b**) status for study medication in trial. AE, adverse event; AG, age group.

 The study was initiated on 8 August 2019, and completed on 25 August 2023. Among 65 participants <18 years of age enrolled in the study, 28 participants were 12 to <18 years of age, 29 participants were 2 to <12 years of age, and 8 participants were <2 years of age (Fig. 2a). Sixty-three participants (96.9%) received at least one dose of letermovir, and 48 (73.8%) completed treatment (Fig. 2a). The most common reasons for early discontinuation of letermovir were AEs (12.7%) and lack of efficacy (i.e., due to CS-CMVi; 12.7%) (Fig. 2b).

 Participant demographics and baseline clinical characteristics are shown in Table 1. Approximately 70% of participants were male, and the median age was 11.0 years (range, 0–17). The most common indications for HCT were acute myeloid leukemia (11 [17.5%] cases), aplastic anemia (6 [9.5%] cases), and chronic granulomatous disease (4 [6.3%] cases) (Table S4). Seven (11.1%) participants had detectable CMV viremia on Day 1 of treatment and were not included in the primary efficacy analysis.

**TABLE 1:** Baseline demographic and clinical characteristics (all participants as treated)^*a*^

Parameter	AG1 (*n* = 28)	AG2 (*n* = 27)	AG3 (*n* = 8)	Total (*N* = 63)
Sex, *n* (%)				
Male	15 (53.6)	22 (81.5)	7 (87.5)	44 (69.8)
Female	13 (46.4)	5 (18.5)	1 (12.5)	19 (30.2)
Age (years)				
Mean (SD)	14.1 (1.5)	6.6 (3.2)	0.7 (0.3)	9.1 (5.3)
Median (range)	13.5 (12–17)	7.0 (2–11)	0.7 (0–1)	11.0 (0–17)
Race, *n* (%)				
Asian	6 (21.4)	3 (11.1)	0	9 (14.3)
Black or African American	3 (10.7)	0	0	3 (4.8)
Multiple	4 (14.3)	2 (7.4)	1 (12.5)	7 (11.1)
White	15 (53.6)	22 (81.5)	7 (87.5)	44 (69.8)
Stem cell source, *n* (%)				
Peripheral blood	15 (53.6)	16 (59.3)	4 (50.0)	35 (55.6)
Bone marrow	12 (42.9)	10 (37.0)	3 (37.5)	25 (39.7)
Cord blood	1 (3.6)	1 (3.7)	1 (12.5)	3 (4.8)
Conditioning regimen used, *n* (%)				
Myeloablative	25 (89.3)	24 (88.9)	6 (75.0)	55 (87.3)
Reduced intensity conditioning	3 (10.7)	1 (3.7)	2 (25.0)	6 (9.5)
Non-myeloablative	0	2 (7.4)	0	2 (3.2)
Days from transplantation to randomization				
Mean (SD)	9.9 (8.38)	9.0 (8.59)	6.5 (4.07)	9.1 (8.03)
Median (range)	7.5 (1.0–28.0)	5.0 (1.0–27.0)	7.5 (1.0–11.0)	7.0 (1.0–28.0)
Donor/recipient CMV serostatus, *n* (%)				
D+/R+	20 (71.4)	18 (66.7)	0	38 (60.3)
D−/R+	8 (28.6)	6 (22.2)	4 (50.0)	18 (28.6)
D+/R−	0	3 (11.1)	4 (50.0)	7 (11.1)

^*a*^ AG, age group; CMV, cytomegalovirus; D+, CMV-seropositive donor; D−, CMV-seronegative donor; R+, CMV-seropositive recipient; R−, CMV-seronegative recipient; SD, standard deviation.

### PK

 The PK analyses were performed on a subset (*n* = 36) of the PK population who underwent intensive PK sampling and did not have any missing PK samples on intensive PK sampling days. The intensive and sparse PK data for each AG (total *n* = 60) were included in the pediatric HCT PopPK analysis, which was used to optimize the final dose recommendations. The pediatric PopPK analysis will be described in a separate publication.

**Fig 3:**
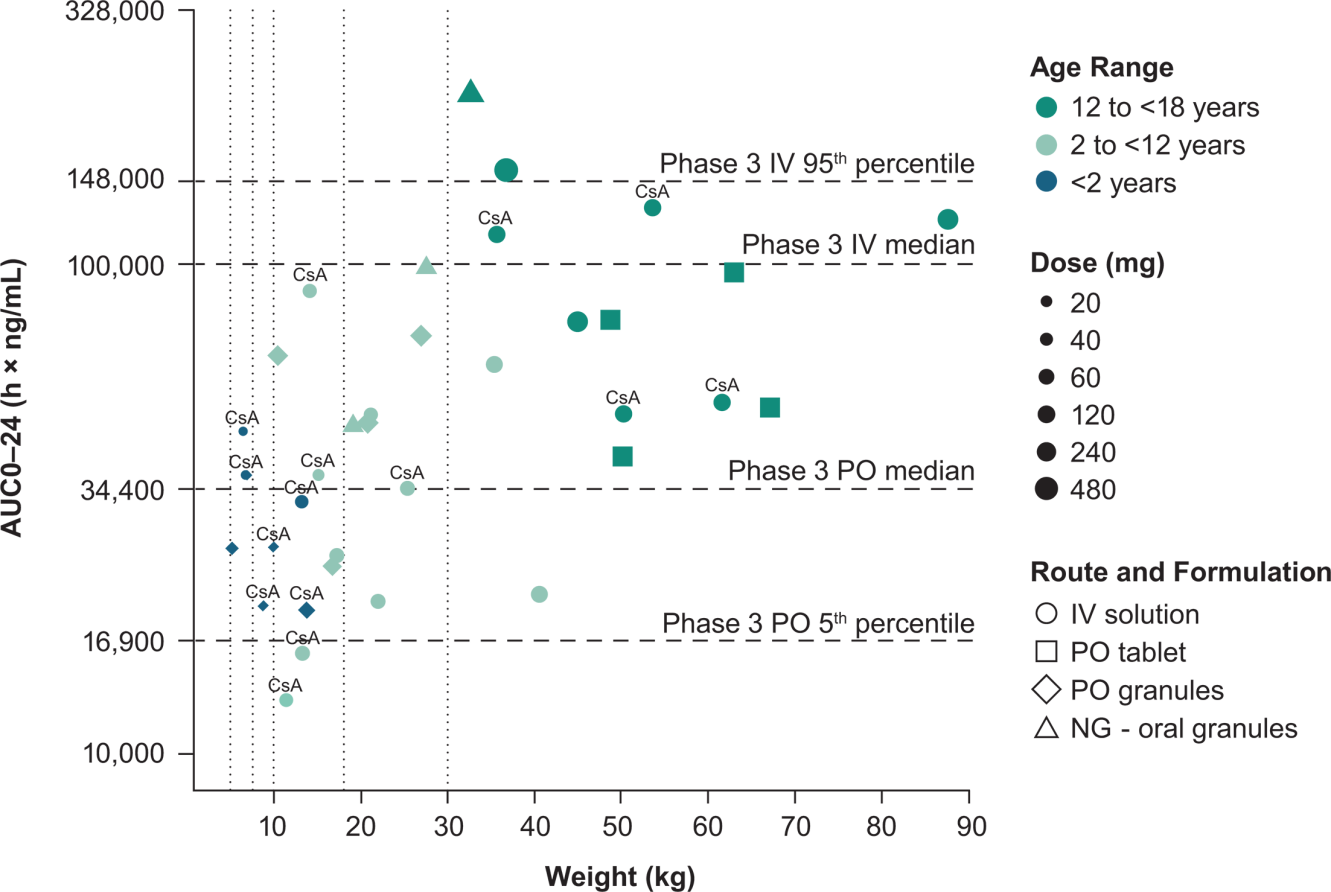
Individual letermovir exposures for all participants who underwent intensive PK sampling compared with reference adult values (*n* = 36). AG, age group; AUC0–24, area under the concentration-time curve for the dosing interval (0–24 h) (logarithmic scale); CsA, cyclosporin A; IV, intravenous; NG, nasogastric; PK, pharmacokinetic; PO, oral; PopPK, population PK. CsA above the symbol indicates CsA was coadministered with the letermovir dose. Vertical dotted lines show the dosing weight-band boundaries for 2 to < 12 years (AG2) and birth to < 2 years (AG3); there are no dosing weight boundaries for 12 to < 18 years (AG1). The upper and lower bounds of the adult reference exposure range were prespecified based on the Phase 3 PopPK model (9).

 Of the 12 PK-evaluable participants in AG1, 8 received letermovir 480 mg orally or IV once daily without CsA, and 4 received letermovir 240 mg IV once daily with CsA (Fig. 3). Participants in AG1 Panel A achieved letermovir exposures comparable with the reference adult exposure, with one participant (480 mg, oral) achieving a higher exposure than the adult reference range, but lower than the maximum observed in the Phase 1 letermovir program (upper limit of 328,000 h × ng/mL). No dose modifications were necessary in AG1 Panel A based on interim PK analysis. All participants in AG1 Panel B achieved letermovir exposures comparable with the reference adult exposure (Fig. 3).

 Of the 16 PK-evaluable participants in AG2, 6 received letermovir 240 mg orally or IV once daily without CsA, 4 received letermovir 120 mg orally or IV once daily without CsA, 1 received letermovir 60 mg IV once daily without CsA, 1 received letermovir 120 mg IV once daily with CsA, and 4 received letermovir 60 mg IV once daily with CsA (Fig. 3). Similar to AG1, participants in AG2 Panel A, all of whom received oral letermovir without concomitant CsA, experienced letermovir exposures comparable with the reference adult exposure. No dose modifications based on the interim PK analysis were necessary in AG2 Panel B. In AG2 Panel B, eight of the participants who received IV letermovir were within the reference adult exposure range, and the remaining two participants, who received IV letermovir with CsA, had exposures lower than the reference adult exposure (Fig. 3).

 Of the eight PK-evaluable participants in AG3, one received letermovir 60 mg orally once daily without CsA, one received letermovir 40 mg IV once daily without CsA, two received letermovir 60 mg orally or IV once daily with CsA, three received letermovir 40 mg orally once daily with CsA, and one received letermovir 20 mg IV once daily with CsA (Fig. 3). Letermovir exposures for the first three AG3 participants, all of whom received concomitant CsA, were lower than the target median adult exposure (Fig. 3). The letermovir dose was increased for the remaining five participants (body weight <10 kg) (Table S3). The resulting letermovir exposures were comparable with the reference adult exposures (Fig. 3).

### Efficacy

Of the 56 participants evaluable for efficacy (i.e., received at least one dose of study drug and had undetectable viremia on Day 1) using the NC = F approach, there were 11 (19.6%) failures (true and imputed) through Week 14 post-HCT and 14 (25.0%) failures through Week 24 post-HCT (Table 2). The proportion of participants for whom treatment failed was similar across the three AGs at both time points, and there were no cases of CMV end-organ disease at either time point.

**TABLE 2:** Proportion of participants with CS-CMVi through Weeks 14 and 24 post-HCT (NC=F approach, primary efficacy population)^*a*^^*,^b^*^

Parameter, *n* (%)	AG1 (*n* = 25)	AG2 (*n* = 24)	AG3 (*n* = 7)	Total (*N* = 56)
Through Week 14 post-HCT				
Failures^*c*^	5 (20.0)	4 (16.7)	2 (28.6)	11 (19.6)
CS-CMVi^*d*^ through visit window	2 (8.0)	1 (4.2)	1 (14.3)	4 (7.1)
Initiation of PET based on documented CMV viremia	2 (8.0)	1 (4.2)	1 (14.3)	4 (7.1)
CMV end-organ disease	0	0	0	0
Discontinued from study before visit window	2 (8.0)	3 (12.5)	1 (14.3)	6 (10.7)
Missing outcome in visit window	1 (4.0)	0	0	1 (1.8)
Through Week 24 post-HCT				
Failures^*c*^	6 (24.0)	6 (25.0)	2 (28.6)	14 (25.0)
CS-CMVi^*d*^ through visit window	2 (8.0)	3 (12.5)	1 (14.3)	6 (10.7)
Initiation of PET based on documented CMV viremia	2 (8.0)	3 (12.5)	1 (14.3)	6 (10.7)
CMV end-organ disease	0	0	0	0
Discontinued from study before visit window	4 (16.0)	3 (12.5)	1 (14.3)	8 (14.3)
Missing outcome in visit window	0	0	0	0

^*a*^ AG, age group; CMV, cytomegalovirus; CS-CMVi, clinically significant CMV infection; HCT, hematopoietic cell transplant; NC = F, non-completer = failure; PET, pre-emptive therapy.

^*b*^ Primary efficacy population, defined as all participants who received ≥1 dose of study intervention and had no detectable CMV viral DNA on Day 1 of treatment.

^*c*^ Categories of failure are mutually exclusive and listed in hierarchical order. With the NC = F approach, failure was defined as all participants who developed CS-CMVi, prematurely discontinued from the study, or had a missing outcome through the post-HCT visit window.

^*d*^ Defined as proven or probable CMV end-organ disease, or initiation of PET based on documented CMV viremia and the participant’s clinical condition.

The true failure rate (incidence of CS-CMVi only) as evaluated using the DAO approach for missing data were 8.2% (4/49) and 12.5% (6/48) at Weeks 14 and 24, respectively (Table S5).

### Safety

There were 63 participants in the safety population (i.e., those who received at least one dose of study drug). Overall, the AE profile was comparable across the three AGs in the broad categories of AEs during the treatment phase (Table S6). The most common AEs reported were vomiting (37 [58.7%] cases), pyrexia (27 [42.9%] cases), diarrhea (24 [38.1%] cases), graft-versus-host disease (GVHD; 24 [38.1%] cases), nausea (18 [28.6%] cases), and abdominal pain (18 [28.6%] cases) (Table 3). Among these, the most common drug-related AEs were gastrointestinal disorders, including vomiting (11 [17.5%] cases) and nausea (2 [3.2%] cases) (Table S7).

**TABLE 3:** Participants with AEs through Week 14 post-HCT (incidence ≥20% in one or more AG; safety population; treatment phase)^*a*^^*,^b^*^

*n* (%)	AG1 (*n* = 28)	AG2 (*n* = 27)	AG3 (*n* = 8)	Total (*N* = 63)
With ≥1 AE^*c*^	28 (100)	27 (100)	8 (100)	63 (100)
Gastrointestinal disorders	27 (96.4)	27 (100)	5 (62.5)	59 (93.7)
Vomiting	14 (50.0)	20 (74.1)	3 (37.5)	37 (58.7)
Nausea	12 (42.9)	6 (22.2)	0	18 (28.6)
Diarrhea	12 (42.9)	12 (44.4)	0	24 (38.1)
Abdominal pain	11 (39.3)	6 (22.2)	1 (12.5)	18 (28.6)
Stomatitis	8 (28.6)	9 (33.3)	0	17 (27.0)
Infections and infestations	21 (75.0)	18 (66.7)	5 (62.5)	44 (69.8)
Device-related infection	1 (3.6)	3 (11.1)	2 (25.0)	6 (9.5)
Epstein-Barr virus infection reactivation	2 (7.1)	0	2 (25.0)	4 (6.3)
General disorders and administration site conditions	16 (57.1)	18 (66.7)	4 (50.0)	38 (60.3)
Pyrexia	11 (39.3)	13 (48.1)	3 (37.5)	27 (42.9)
Immune system disorders	15 (53.6)	13 (48.1)	4 (50.0)	32 (50.8)
Graft-versus-host disease	10 (35.7)	11 (40.7)	3 (37.5)	24 (38.1)
Skin and subcutaneous tissue disorders	12 (42.9)	17 (63.0)	2 (25.0)	31 (49.2)
Pruritus	6 (21.4)	3 (11.1)	0	9 (14.3)
Metabolism and nutritional disorders	12 (42.9)	14 (51.9)	4 (50.0)	30 (47.6)
Decreased appetite	3 (10.7)	8 (29.6)	0	11 (17.5)
Blood and lymphatic system disorders	8 (28.6)	15 (55.6)	1 (12.5)	24 (38.1)
Thrombocytopenia	5 (17.9)	9 (33.3)	0	14 (22.2)
Neutropenia	2 (7.1)	8 (29.6)	1 (12.5)	11 (17.5)
Anemia	1 (3.6)	7 (25.9)	0	8 (12.7)
Vascular disorders	10 (35.7)	11 (40.7)	2 (25.0)	23 (36.5)
Hypertension	7 (25.0)	9 (33.3)	0	16 (25.4)
Renal and urinary disorders	14 (50.0)	8 (29.6)	0	22 (34.9)
Dysuria	6 (21.4)	3 (11.1)	0	9 (14.3)
Nervous system disorders	13 (46.4)	5 (18.5)	1 (12.5)	19 (30.2)
Headache	6 (21.4)	3 (11.1)	0	9 (14.3)

^*a*^ AE, adverse event; AG, age group; HCT, hematopoietic cell transplant.

^*b*^ Safety population, defined as all participants who received ≥1 dose of study drug.

^*c*^ AEs were reported using the Medical Dictionary for Regulatory Activities Version 25.1. Each participant was counted once for each system organ class or specific AE.

Thirty-five (55.6%) participants experienced at least one SAE (Table S6). The most commonly reported SAEs were pyrexia (7.9%) and GVHD (4.8%). Only two participants had SAEs that were considered by investigators to be drug-related (one case each of atrial fibrillation and increased blood bilirubin). Both SAEs were multifactorial in origin, such that other etiologies could not be ruled out, and both resolved.

There were four deaths reported during the treatment phase that were considered to be due to AEs. The causes of death included *Candida* infection and multiple organ dysfunction syndrome (*n* = 1), post-HCT lymphoproliferative disorder and hepatosplenic candidiasis (*n* = 1), recurrent acute myeloid leukemia (*n* = 1), and septic shock (*n* = 1). None of the deaths were considered related to the study drug by the investigators.

### Viral resistance

Resistance genotyping was attempted on samples collected from 12 participants with viremia. Of these, 10 participants had evaluable sequence data available for analysis (AG1, *n* = 4; AG2, *n* = 4; and AG3, *n* = 2). In two participants, known letermovir resistance-associated amino acid substitutions were detected in pUL56 at an allele frequency above the validated assay cutoff of 5%. One was an AG1 participant who had an R369S substitution detected on Day 54 of the study. The other, an AG3 participant who was positive for CMV DNAemia on Day 1, had a C325W substitution detected on Day 129 of the study. Among the 10 participants with evaluable data, no known letermovir resistance-associated substitutions were observed in the pUL51 or pUL89 terminase subunits.

## DISCUSSION

Pediatric allogeneic HCT recipients are at risk of CMV infection or reactivation (1, 4), and there is a significant need for an antiviral agent for CMV prophylaxis in this population (4). As the pathogenesis and manifestations of CMV infection and disease are similar in adult and pediatric patients (11), the initial dose selections and dose recommendations of letermovir were based on achieving comparable exposures anticipated to result in similar efficacy and safety in both populations. The primary objective of this study was to evaluate the PK of letermovir in pediatric HCT recipients from birth to <18 years of age to inform proposed pediatric dosing of letermovir. Another objective of the study was to generate additional data to provide substantial evidence for the safe and effective use of letermovir in this population. Viral resistance was also assessed in this study.

The doses used in this study were based on simulations using an allometric scaling of an adult HCT PopPK model to approximate letermovir exposures in pediatric participants to the adult HCT reference exposure range. There were no letermovir dose modifications to the initial dose selections for AG1 and AG2. For AG3, the youngest participants, letermovir exposures for the first three participants (who received letermovir with CsA) were trending lower than the target median adult exposure at the interim analysis. In the remaining five AG3 participants, the letermovir dose was increased for participants weighing <10 kg. The letermovir exposures in pediatric participants for the doses evaluated in the study were generally within the adult HCT recipient reference exposure range.

The final dose recommendations for the pediatric HCT population were based on using the PK data from this study in a pediatric HCT PopPK model, which will be the subject of a separate publication. The predicted letermovir exposures, based upon the final pediatric HCT PopPK model, informed and supported the dose recommendations of the recent pediatric approval (7). The weight-band dosing of letermovir for participants <12 years of age is largely consistent with the doses evaluated in this study with the addition of combining some weight bands across ages.

The efficacy of letermovir in preventing CS-CMVi in this study was consistent with the efficacy demonstrated in the pivotal Phase 3 adult study. With the NC = F approach used to account for missing data, 19.6% of study participants had failed at Week 14 post-HCT, compared with 19.1% in the adult study at the same time point. The corresponding rates at Week 24 post-HCT were 25.0% and 37.5% in pediatric and adult participants, respectively (6).

It should be noted that the NC = F approach for estimating failure rates is a conservative approach, where the failure rate includes not only individuals who develop CS-CMVi (true failures), but also those who discontinue the study prematurely or where data are missing (imputed failures). The true failure rate with the DAO approach was substantially lower (8.2% and 12.5% at Weeks 14 and 24 post-HCT, respectively).

It should also be noted that a subset of participants from AG2 and AG3 were CMV-seronegative recipients with a CMV-seropositive donor (D+/R−) at baseline (to facilitate study enrollment of the younger AGs given the relatively small number of HCTs performed in pediatric patients and lower CMV seroprevalence observed globally in younger individuals [3, 4]). As D+/R− allogeneic HCT recipients have a lower risk of developing CS-CMVi (~30% risk [12]), this would be a potential caveat when comparing failure rates in this study with the adult data.

Letermovir was generally safe and well tolerated in this study, with a safety profile similar to that seen in the adult Phase 3 HCT study. Gastrointestinal-related AEs were the most common treatment-emergent AEs, and treatment discontinuation due to drug-related AEs was rare (3.2%; Table S6). No treatment-related deaths were reported. Thus, letermovir has a favorable toxicity profile compared with other anti-CMV agents (such as ganciclovir, foscarnet, and cidofovir); most notably, letermovir shows no evidence of hematologic toxicity in the allogeneic HCT setting (1, 6, 13).

There are several well-recognized approaches to providing substantial evidence for the safe and effective use of a drug in a pediatric population that often involve leveraging available data in adults. This concept is referred to as pediatric extrapolation (14, 15), and it is an accepted means to facilitate pediatric drug approval without necessitating large pediatric Phase 3 trials. Pediatric extrapolation of efficacy is considered to be appropriate when it can be assumed that the course of the disease and the expected response to a drug would be sufficiently similar in the pediatric and reference populations.

For the purpose of pediatric extrapolation, robust safety and efficacy data were available from a large multicenter trial in adults demonstrating a clinically meaningful and statistically significant effect on CS-CMVi prevention, as well as a mortality benefit (6). Confirmation of these results in a pediatric population in a second large trial with a traditional design (i.e., large sample size, double-blind with a placebo comparator) was unnecessary and would have been impracticable given the difficulties of patient recruitment for pediatric trials and the length of time needed to complete them (16). A traditional trial design would also have presented significant ethical concerns as no additional benefits would have been demonstrated, and there would have been substantial delays in the availability of a much-needed intervention in this vulnerable patient population.

The PK data obtained in this study were used in a pediatric HCT PopPK model to predict exposures for this population and support proposed dose recommendations, along with additional safety and efficacy data supporting pediatric extrapolation, for submission to regulatory authorities. The first regulatory approval for use of letermovir in pediatric R+ HCT recipients for CMV prophylaxis was obtained in August 2024. Final dose recommendations are provided in the patient prescribing information (Table S8).

In conclusion, at the doses used and the letermovir exposures achieved in this study, letermovir is generally safe and effective in preventing CS-CMVi in pediatric HCT recipients.

## Data Availability

The data sharing policy, including restrictions, of Merck Sharp & Dohme LLC, a subsidiary of Merck & Co., Inc., Rahway, NJ, USA, is available at https://externaldatasharing-msd.com/. Requests for access to the clinical study data can be submitted via email to the Data Access mailbox (dataaccess@msd.com).

## SUPPLEMENTAL MATERIAL

Supplemental tables aac.00420-25-s0001.pdf Tables S1 to S8.

ASM does not own the copyrights to Supplemental Material that may be linked to, or accessed through, an article. The authors have granted ASM a non-exclusive, world-wide license to publish the Supplemental Material files. Please contact the corresponding author directly for reuse.
