# Supplementary material for: Cytomegalovirus prophylaxis with letermovir in pediatric (birth to <18 years of age) hematopoietic cell transplant recipients: pharmacokinetics, efficacy, and safety results of a Phase 2b study
Source: Antimicrob Agents Chemother. 2025 Aug 18;69(10):e00420-25. doi: 10.1128/aac.00420-25 (PMC12486802; doi:10.1128/aac.00420-25)
Supplement: Supplemental tables — Tables S1 to S8. [file aac.00420-25-s0001.pdf]

**Supplementary Material**

***Supplementary Table S1. Inclusion criteria***

| |
|---|
| Participant is aged from birth to <18 years of age at the time of signing the informed consent/assent |
| All AG1 participants must have documented positive CMV serostatus (CMV IgG seropositive) for the recipient (R+) within 90 days prior to enrollment<br><br>Participants in AG2 and AG3 must have documented positive CMV serostatus (CMV IgG seropositive) for the recipient (R+) within 90 days prior to enrollment and/or for the donor (D+); the donor serostatus should be documented within 1 year prior to enrollment |
| Be the recipient of a first allogeneic HCT (bone marrow, peripheral blood stem cell, or cord blood transplant) within 28 days prior to enrollment |
| Have undetectable CMV DNA from a plasma or whole blood sample collected within 5 days prior to enrollment |
| Female participants were eligible if they were not pregnant or breastfeeding, not a woman of childbearing potential, or, if of child-bearing potential, agreed to use appropriate contraception during the treatment period and for 90 days after the last treatment dose |
| The participant (or legally acceptable representative) has provided documented informed consent/assent for the study |
| Study participants in Panel A of AG1 and AG2 must not be on concomitant CsA and must be able to take (as assessed by the investigator) letermovir tablets or oral granules (either by mouth or via gastric or nasogastric tube), provided the participant does not have a condition that may interfere with the absorption of oral medication (e.g., vomiting, diarrhea, or a malabsorptive condition) from the day of enrollment until the intensive PK sampling is completed in these panels) |
| For AG2, the participant's weight should be ≥10 kg; for AG3, the participant's weight should be<br>≥2.5 kg and ≤15 kg at the time of enrollment |

AG, age group; CMV, cytomegalovirus; CsA, cyclosporin A; HCT, hematopoietic cell transplant; IgG, immunoglobulin G.

***Supplementary Table S2. Exclusion criteria***

| |
|---|
| Prior allogeneic HCT |
| CMV end-organ disease within 6 months prior to enrollment |
| Evidence of CMV viremia at any time between informed consent or HCT procedure (whichever was earlier) until enrollment |
| Suspected or known hypersensitivity to active or inactive ingredients of letermovir formulations |
| Severe hepatic insufficiency, defined as Child-Pugh Class C, within 5 days prior to enrollment |
| Serum aspartate aminotransferase or alanine aminotransferase >5× the ULN or serum total bilirubin >2.5× ULN within 5 days prior to enrollment |
| Receiving renal replacement therapy or has end-stage renal impairment with creatinine clearance<br>≤10 mL/min (aged ≥12 years) or ≤10 mL/min/1.73 $m^2$ (aged <12 years) using serum creatinine within<br>5 days prior to enrollment |
| Has both moderate hepatic insufficiency (Child-Pugh Class B) and moderate-to-severe renal insufficiency (<50 mL/min for AG1; <50 mL/min per 1.73 $m^2$ for AG2; <20 mL/min per 1.73 $m^2$ for AG3) |
| Has an uncontrolled infection, is on mechanical ventilation, or is hemodynamically unstable on day of enrollment |
| Has a history of HIV positivity at any time, or positivity for hepatitis C or hepatitis B within 90 days prior to enrollment |
| Has an active solid tumor malignancy, except localized basal cell or squamous cell skin cancer or the condition under treatment |
| Has a preexisting cardiac condition requiring current treatment or resulting in hospitalization within prior 6 months or expected to recur during the study |
| Exposure to ganciclovir, valganciclovir, foscarnet, acyclovir (at doses greater than those recommended for HSV/VZV prophylaxis), or valacyclovir (at doses greater than those recommended for HSV/VZV prophylaxis), or famciclovir within 7 days prior to enrollment |
| Exposure to cidofovir, CMV immunoglobulin, any investigational CMV antiviral agent/biologic therapy, rifampin and other strong inducers (such as phenytoin, carbamazepine, St John's |

| |
|---|
| wort, rifabutin, and phenobarbital) and moderate inducers (such as nafcillin, thioridazine, modafinil, and bosentan) within 30 days prior to enrollment |
| Previous exposure to letermovir |
| Currently participating or has participated in a study with an unapproved investigational compound or device within 28 days, or 5× the half-life of the investigational compound (excluding monoclonal antibodies), whichever is longer, of initial dosing in this study. Participants previously treated with a monoclonal antibody will be eligible to participate after a 28-day washout period |
| Has previously participated in this study or any other study involving letermovir, or has previously participated or is currently participating in any study involving administration of a CMV vaccine or another CMV investigational agent, or is planning to participate in a study of a CMV vaccine or another CMV investigational agent during the course of this study |
| Is pregnant or expecting to conceive, is breastfeeding, or plans to breastfeed from the time of consent through 28 days after the last dose of study intervention, or is expecting to donate eggs starting from the time of consent through 28 days after the last dose of study intervention |
| Has clinically relevant drug or alcohol abuse within 12 months of screening that may interfere with participant treatment, assessment, or compliance with the protocol, as assessed by the investigator; or has a history or current evidence of any condition, therapy, laboratory abnormality, or other circumstance that might confound the results of the study, interfere with the participant's participation for the full duration of the study, or would be put at undue risk as judged by the investigator; or is or has an immediate family member who is investigational site or Sponsor staff directly involved with this study |

AG, age group; CMV, cytomegalovirus; CsA, cyclosporin A; HCT, hematopoietic cell transplant; HSV, herpes simplex virus; IgG, immunoglobulin G; ULN, upper limit of normal; VZV, varicella zoster virus.

*Supplementary Table S3. Letermovir doses used in the study*

| Age group | Body weight limits (kg) | Oral letermovir dose (mg) | Oral letermovir dose with CsA (mg) | IV letermovir dose[a] (mg) | IV letermovir dose with CsA[b] (mg) |
|---|---|---|---|---|---|
| AG1 | Any weight | 480 | 240 | 480 | 240 |
| AG2 | ≥30 | 480 | 240 | 240 | 240 |
| | 18 to <30 | 240 | 120 | 120 | 120 |
| | 10 to <18 | 120 | 60 | 60 | 60 |
| AG3 (first 3 participants) | 10 to ≤15 | 120 | 60 | 60 | 60 |
| | 7.5 to <10 | 80 | 40 | 40 | 40 |
| | 5.0 to <7.5 | 40 | 20 | 20 | 20 |
| | 2.5 to <5.0 | 20 | 10 | 10 | 10 |
| AG3 (last 5 participants)[c] | 10 to ≤15 | 120 | 60 | 60 | 60 |
| | 7.5 to <10 | 120 | 60 | 60 | 60 |
| | 5.0 to <7.5 | 60 | 40 | 40 | 40 |
| | 2.5 to <5.0 | 40 | 20 | 20 | 20 |

AG, age group; CsA, cyclosporin A; IV, intravenous.

[a]Based on modeling, for AG2 and AG3, the IV dose of letermovir without CsA was reduced by 50% compared with oral letermovir in order to maintain target exposures.

[b]No further reduction of IV letermovir was necessary for AG2 and AG3 when coadministered with CsA.

[c]Based on interim analysis results for AG3, doses were increased for the last 5 participants weighing <10 kg.

*Supplementary Table S4. Most common[a] conditions necessitating transplant (all participants as treated)*

| Parameter, n (%) | AG1 (n=28) | AG2 (n=27) | AG3 (n=8) | Total (N=63) |
|---|---|---|---|---|
| Acute myeloid leukemia | 6 (21.4) | 5 (18.5) | 0 | 11 (17.5) |
| Aplastic anemia | 4 (14.3) | 2 (7.4) | 0 | 6 (9.5) |
| Chronic granulomatous disease | 1 (3.6) | 3 (11.1) | 0 | 4 (6.3) |
| Acute lymphocytic leukemia | 0 | 2 (7.4) | 1 (12.5) | 3 (4.8) |
| Acute lymphocytic leukemia recurrent | 3 (10.7) | 0 | 0 | 3 (4.8) |
| B precursor type acute leukemia | 2 (7.1) | 1 (3.7) | 0 | 3 (4.8) |
| Combined immunodeficiency | 0 | 0 | 3 (37.5) | 3 (4.8) |
| Familial hemophagocytic lymphohistiocytosis | 0 | 1 (3.7) | 2 (25.0) | 3 (4.8) |
| B-cell type acute leukemia | 1 (3.6) | 1 (3.7) | 0 | 2 (3.2) |
| Myelodysplastic syndrome | 1 (3.6) | 1 (3.7) | 0 | 2 (3.2) |
| Sickle cell disease | 1 (3.6) | 1 (3.7) | 0 | 2 (3.2) |

AG, age group.

[a]Reported by ≥2 participants in the overall study population.

***Supplementary Table S5. Proportion of participants with CS-CMVi through Week 14 and Week 24 post-HCT (DAO approach, primary efficacy population[a])***

| **Parameter, n (%)** | **AG1** | **AG2** | **AG3** | **Total** |
|---|---|---|---|---|
| Through Week 14 post-HCT, N | 22 | 21 | 6 | 49 |
| Failures[b] | 2 (9.1) | 1 (4.8) | 1 (16.7) | 4 (8.2) |
| CS-CMVi[c] through visit window | 2 (9.1) | 1 (4.8) | 1 (16.7) | 4 (8.2) |
| Initiation of PET based on documented CMV viremia | 2 (9.1) | 1 (4.8) | 1 (16.7) | 4 (8.2) |
| CMV end-organ disease | 0 | 0 | 0 | 0 |
| Through Week 24 post-HCT, N | 21 | 21 | 6 | 48 |
| Failures[b] | 2 (9.5) | 3 (14.3) | 1 (16.7) | 6 (12.5) |
| CS-CMVi[c] through visit window | 2 (9.5) | 3 (14.3) | 1 (16.7) | 6 (12.5) |
| Initiation of PET based on documented CMV viremia | 2 (9.5) | 3 (14.3) | 1 (16.7) | 6 (12.5) |
| CMV end-organ disease | 0 | 0 | 0 | 0 |

AG, age group; CMV, cytomegalovirus; CS-CMVi, clinically significant CMV infection; DAO, data as observed; HCT, hematopoietic cell transplant; PET, pre-emptive therapy.

[a]Primary efficacy population, defined as all allocated participants who received ≥1 dose of study intervention and had no detectable CMV viral DNA on Day 1 of treatment.

[b]Categories of failure are mutually exclusive and listed in hierarchical order. With the DAO approach, any participant with a missing value for a particular endpoint was excluded from the analysis.

[c]Defined as proven or probable CMV end-organ disease, or initiation of PET based on documented CMV viremia and the participant's clinical condition.

***Supplementary Table S6. AE summary through Week 14 (treatment phase); safety population[a]***

| AE, n (%) | AG1 (n=28) | AG2 (n=27) | AG3 (n=8) | Total (N=63) |
|---|---|---|---|---|
| With ≥1 AE | 28 (100) | 27 (100) | 8 (100.0) | 63 (100) |
| Drug-related[b] AEs | 9 (32.1) | 8 (29.6) | 3 (37.5) | 20 (31.7) |
| Serious AEs | 12 (42.9) | 17 (63.0) | 6 (75.0) | 35 (55.6) |
| Serious drug-related AEs | 2 (7.1) | 0 | 0 | 2 (3.2) |
| Death[c] | 3 (10.7) | 1 (3.7) | 0 | 4 (6.3) |
| Discontinued due to: | | | | |
| AE | 5 (17.9) | 2 (7.4) | 1 (12.5) | 8 (12.7) |
| Drug-related AE | 2 (7.1) | 0 | 0 | 2 (3.2) |
| Serious AE | 2 (7.1) | 2 (7.4) | 1 (12.5) | 5 (7.9) |
| Serious drug-related AE | 1 (3.6) | 0 | 0 | 1 (1.6) |

AE, adverse event; AG, age group.

[a]Safety population, defined as all participants who received ≥1 dose of study drug.

[b]Determined by the investigator to be related to the drug.

[c]None of the deaths were considered drug-related.

***Supplementary Table S7. Participants with drug-related AEs through Week 14 post-HCT (incidence ≥20% in one or more age group; all participants as treated; treatment phase)***

| **n (%)** | **AG1 (n=28)** | **AG2 (n=27)** | **AG3 (n=8)** | **Total (N=63)** |
|---|---|---|---|---|
| With ≥1 drug-related AE[a] | 9 (32.1) | 8 (29.6) | 3 (37.5) | 20 (31.7) |
| **Gastrointestinal disorders** | **4 (14.3)** | **5 (18.5)** | **2 (25.0)** | **11 (17.5)** |
| Vomiting | 4 (14.3) | 5 (18.5) | 2 (25.0) | 11 (17.5) |
| Nausea | 1 (3.6) | 1 (3.7) | 0 | 2 (3.2) |

AE, adverse event; AG, age group; HCT, hematopoietic cell transplant.

[a]AEs were reported using the Medical Dictionary for Regulatory Activities Version 25.1. Each participant was counted once for each system organ class or specific AE.

***Supplementary Table S8. Dose recommendations for letermovir in pediatric HCT recipients in the United States aged 6 months to <12 years or ≥12 years and weighing <30 kg[a]***

| **Body weight (kg)** | **Daily oral letermovir dose (mg)** | **Daily oral letermovir dose with CsA (mg)** | **Daily IV letermovir dose (mg)** | **Daily IV letermovir dose with CsA (mg)** |
|---|---|---|---|---|
| ≥30 | 480 | 240 | 480 | 240 |
| 15 to <30 | 240 | 120 | 120 | 120 |
| 7.5 to <15 | 120 | 60 | 60 | 60 |
| 6 to <7.5 | 80 | 40 | 40 | 40 |

CsA, cyclosporin A; HCT, hematopoietic cell transplant; IV, intravenous.

[a]Listed dose recommendations are per the US prescribing information, revised January 2025 (Prevymis [package insert]. Merck Sharp & Dohme LLC; 2025). Recommendations may vary by country due to differences in weight bands, and clinicians should refer to their appropriate country-specific labels.
